# Host-Microbe Interaction on the Skin and Its Role in the Pathogenesis and Treatment of Atopic Dermatitis

**DOI:** 10.3390/pathogens11010071

**Published:** 2022-01-06

**Authors:** Danuta Nowicka, Karolina Chilicka, Iwona Dzieńdziora-Urbińska

**Affiliations:** 1 Department of Dermatology, Venereology and Allergology, Wroclaw Medical University, 50-368 Wrocław, Poland; 2 Department of Health Sciences, University of Opole, 45-040 Opole, Poland; karolina.chilicka@uni.opole.pl (K.C.); iwona.dziendziora@uni.opole.pl (I.D.-U.)

**Keywords:** microbiome, atopic dermatitis, skin colonization, skin infection

## Abstract

Atopic dermatitis (AD) is a condition with a complex and unclear aetiology. Possible causes of AD encompass alterations in the structure and function of the epidermal barrier, disturbances in the skin microbiome, immune factors, allergens, bacterial and fungal infections as well as environmental and genetic factors. In patients with AD, acute skin lesions are colonized by a greater number of bacteria and fungi than chronic lesions, clinically unchanged atopic skin and the skin of healthy people. Mechanisms promoting skin colonization by pathogens include complex interplay among several factors. Apart from disturbances of the skin microbiome, increased adhesion in atopic skin, defects of innate immune response resulting in the lack of or restriction of growth of microorganisms also contribute to susceptibility to the skin colonization of and infections, especially with *Staphylococcus aureus*. This review of the literature attempts to identify factors that are involved in the pathogenesis of AD-related bacterial and fungal skin colonization. Studies on the microbiome, commensal microorganisms and the role of skin microorganisms in maintaining healthy skin bring additional insight into the treatment and prevention of AD. In the light of presented mechanisms, reduction in colonization may become both causative and symptomatic treatment in AD.

## 1. Introduction

Atopic dermatitis (AD) affects approximately 15–20% of children and 1–3% of adults globally, but its aetiology has not been fully explained. The first mentions of AD-like conditions date back to ancient times, but prurigo and prurigo-like conditions were first described in the literature in 1808 by an English physician Robert Willan who proposed the first structured classification of skin diseases [1]. However, the term ‘atopy’ was introduced many years later, in 1923, by Arthur Coca and Robert Cooke. Subsequent observations and experience lead to the introduction of the term ‘atopic dermatitis’ in 1933 by Fred Wise and Marion Sulzberger [2,3].

Although symptoms and causes of AD as well as their evolution in the course of the disease have been widely investigated and described in the literature, the discussion is still ongoing on the entire picture of this disease. Researchers agree that many causative factors contribute to the development of AD, including structural and functional defects of the epidermal barrier, imbalance of the skin microbiome, and disturbed immunity as well as environmental (e.g., allergens) and genetic factors [4]. Many authors consider infections important, particularly focusing on *Staphylococcus aureus* (*S. aureus*) [5,6,7]. The prevalence of *S. aureus* colonization on the non-lesional skin reaches 40%and doubles when lesions develop [6]. Recent reports suggest that colonization of microorganisms can be regarded both as a causative factor and a consequence of a cascade of symptoms and disorders in AD [8].

The course of AD is chronic with variable presentations. Typically, symptoms appear in children up to 3 months of age. About 60% of AD cases are diagnosed in children up to 1 year of age, and 90% up to 5 years of age. In the adult population, women are affected more frequently than men. The clinical picture of AD depends on the phase of the disease which is commonly classified into infant, childhood, adolescent, and adult phase. Itch is a predominant symptom in every type and phase of AD and forces patients to uncontrolled scratching. Generalized lesions are seen in patients with the most severe course of AD [9,10,11].

## 2. The Role of the Epidermal Barrier

An elevated immune and non-specific inflammatory response in the skin of AD patients seems to be triggered by altered skin barrier structure as well as its altered functional integrity and decreased ability for self-renewal [12,13]. Furthermore, defects of the skin barrier make it easier for microorganisms to colonize the skin [14]. The basic role of the skin barrier is to protect against water loss from the epidermis and its underlying layers, minimize penetration of potentially harmful substances and pathological microorganisms, and safeguard against environmental factors (e.g., warmth or cold). Thus, proper functioning of the skin ensures a good condition of the epidermal barrier and a healthy look of the skin. Conversely, defects in the structure of the epidermal barrier which translate into its altered functioning are characteristic for many dermatological diseases, including AD [13,15].

The basic components of the epidermal barrier are corneocytes (stratum corneum), lipids, and natural moisturizing factor (NMF)produced during the maturation of corneocytes. Corneocytes are formed from keratinocytes, which proliferate in the basal layer of the epidermis and during their maturation migrate from the basal layer upwards, toward the outer layers of the skin. These cells are “dead”, flattened, and anucleated. During the first step of cornification, the formation of an intracellular keratin network takes place [16]. Next, they lose the cell nucleus and organelles [17]. Furthermore, the expression of superficial proteins changes from keratin 5 and 14 into 1 and 2e as well as keratin 10. The final differentiation process is facilitated by filaggrin due to its keratin binding ability. Filaggrin is the second most abundant protein in the topmost layers of the epidermis. Degradation of filaggrin increases the amount of NMF and helps to restore water content [18].

As filaggrin, a filament-associated protein, plays an important role in the maintenance of the skin barrier function, mutations within the filaggrin gene leading to the loss of its function are considered a risk factor for AD, atopic eczema, food allergies, and bronchial asthma [19,20,21]. This is due to the fact that a cornified envelope composed mainly of filaggrin, loricrin, trichohyalin, involucrin, and intermediate keratin filaments is built during the terminal differentiation of keratinocytes. This protein envelope makes the epidermal barrier resistant to lytic enzymes [22]. Amino acids and other substances that are released during filaggrin degradation are then used to produce NMF. Apart from free amino acids, mainly salts of pyroglutamic acid, NMF contains urea and inorganic salts. It forms a structure that allows absorbing and binding of water in the protective layer of the epidermis. The stratum corneum of the epidermis contains only approximately 20% of NFM, however, its adequate functioning is due to the unique constituents. Together with the release of amino acids, lipids of the extracellular matrix are synthesized so that ceramides, free fatty acids, cholesterol, and their esters are produced. The extracellular matrix can contain up to 50% of ceramides that are a key for barrier homeostasis [16,23]. Cholesterol is another element of the stratum corneum with a 25% share in the extracellular matrix. Although it can be absorbed from circulation by cells of the basal layer, it is almost completely synthesised in the epidermis [24,25]. 

Changes in the composition of the epidermis may increase the risk of greater retention of microorganisms on the surface of the skin. Long-chain unsaturated free fatty acids serve as protection against colonization and infection by *S. aureus* [26]. However, in AD patients, the change in the lipid and fatty acids fractions in the skin along with a reduced number of ceramides and an elevated amount of cholesterol in the stratum corneum seems to facilitate bacterial colonization [27,28]. The study by Heczko et al. focused on colonization by *S. aureus*. They reported that an insufficient amount of medium-chain-length fatty acids in the epidermis promote bacterial colonization while the opposite effect was observed in the presence of elevated concentrations of capric and lauric acid [29].

New evidence gained on the role of the microbiome has confirmed the role of microorganisms residing on the surface of the skin in preserving the intact skin barrier and defence against pathogens [30,31,32]. Commensal bacteria can mediate the host’s immune responses. They participate in continuous crosstalk with the keratinocytes and the immune system. Langerhans cells, considered skin dendritic cells, elongate their dendrites between keratinocytes creating a dense network with is in close contact with antigens, microbiome and pathogens. Activated Langerhans cells access tight junctions that guard paracellular leakage of water and electrolytes, but in addition, serve as a communication measure [32]. In addition, they maintain immune homeostasis by teaching the host how to recognize and fight against pathogens. Moreover, the host’s microbiome serves as a physiological barrier against pathogens because commensal bacteria occupy and compete for the niches. Microorganisms may be present in a sessile form or form complex multispecies communities within the biofilms. Although reports from the literature show that bacterial biofilms serve as the primary pathogenic factor in a variety of skin diseases, e.g., in acne vulgaris and chronic wounds [33], they are also important to maintain the skin barrier in AD patients.

## 3. The Microbiome and Its Role in Atopic Skin Inflammation

The skin is one of the outermost organs of the human body, the primary function of which is protection against environmental factors. A characteristic feature of its structure is the microbiome consisting of bacteria, fungi, mites and viruses. However, in order for the microbiome to fulfil its specific functions, it must be, above all, characterized by diversity [34]. Human skin has also many features that significantly affect the species and quantitative composition of the microbiome, such as the thickness of its individual layers, the distribution of appendages, as well as moisture and temperature on its surface [34,35,36]. The composition of the skin microbiome in terms of their species has not been fully understood and the reason for incomplete knowledge about these microorganisms are challenges faced during their examination. The skin microbiome varies by location as well as changes in response to environmental factors, skin condition and temporal shifts in the skin homeostasis [34,37,38]. In terms of structure and biology, the skin is extremely hostile to the development of microorganisms. On the surface of the epidermis, the skin is dry and peels off, therefore microorganisms are regularly removed from its surface, so they cannot grow and create a long-lasting biofilm [14]. It should also be mentioned that the presence of a hydrolipid mantle on the skin surface also hinders the growth of microorganisms, as it contains compounds with antibacterial activity, such as lysozyme, dermcidin and sebum [34,36,39,40,41,42]. The keratinocytes, sebocytes, mast cells and sweat gland cells also have the ability to secrete antimicrobial factors. Research shows that there are over 20 antimicrobial peptides on the outer layers of the skin. The factors described above mean that the skin can be inhabited only by certain species of microorganisms and affect their number [43].

Bacterial genomic sequence data in addition to traditional culture-based methods allow investigating the microbial landscape of the healthy skin and skin lesions. Human skin is inhabited mainly by the following bacteria: *Actinobacteria* (*Corynebacterium* spp., including *C. jeikeium; Propionibacterium* spp., including *P. acnes; Microbacterium* spp.; *Micrococcus* spp., including *M. luteus, M. varians, M. lylae, M. sedentarius, M. roseus, M. kristinae* and *M. nishinomiyaensis*), *Firmicutes* non-haemolytic aerobic and anaerobic, staphylococci (*Staphylococcus* spp., including *S. epidermidis, S. saprophyticus, S. hominis, S. warneri, S. haemolyticus* and *S. capitis*), α-hemolytic streptococci (*Streptococcus* spp.), enterococci (*Enterococcus*), *Bacteroidetes* (*Sphingobacterium* spp., *Chryseobacterium* spp.), *Proteobacteria* (*Janthinobacterium* spp., *Serratia* spp., *Halomonas* spp., *Delftia* spp., *Comamonas* spp.) [37,44]. The skin may also be colonized by pathogenic bacteria: group A streptococci (*S. pyogenes*), *Golden Staphylococcus* (*S. aureus*), Gram (-) bacilli (*P. aeruginosa*), and also by aerobic coryneform bacteria (*Corynebacterium* spp.), which are rather commensal microbes, but can cause an infection as well [39,45].

The fungi inhabiting the skin include those of the genus *Malassezia* (*M. furfur, M. sympodialis, M. globosa, M. restricta, M. slooffiae, M. yamatoensis, M. obtusa, M. dermatis* and *M. japonica*). Under favourable conditions and with a lowered immune system response, fungi, such as bacteria, can cause dermatological diseases. The most common diseases caused by the fungi of the genus *Malassezia* include pityriasis versicolor, inflammation of the hair follicles (folliculitis) [44,46,47,48,49]. On human skin, we can also find fungi of the genus *Penicillium* (*P. chrysogenum, P. lanosum*), *Aspergillus* (*A. candidus, A. terreus, A. versicolor*), *Candida* (*C. tropicalis, C. parapsilosis, C. orthopsilosis*), *Chaetomium, Chrysosporium, Cladosporium, Mucor, Debaryomyces, Cryptococcus* (*C. flavus, C. dimmennae, C. diffluent*), *Trichophyton* and *Rhodotorula*, dermatophytes (*Microsporum, Epidermophyton, Trichophyton*) [44,49].In addition to bacteria and fungi, the skin microbiota also includes *Demodex*–mites inhabiting the hair follicles and sebaceous glands. The most numerous representatives are *D. folliculorum* (hair follicles) and *D. brevis* (sebaceous and meibomian glands) [50].

Due to the diversity of viruses in terms of genetic material (DNA, RNA), their transient nature and short-term survival on the skin, their identification and isolation on the skin surface is still a challenge, as it is difficult to create genomic libraries. These particles do not have conserved regions present in the genomes of bacteria or fungi, but it is assumed that viruses are not only a pathogen, but also play an important role in maintaining skin homeostasis. It turns out that they constitute an unstable but important element of the skin microbiome, especially polyomaviruses (*Polyomaviridae*) and papillomaviruses (*Papillomaviridae*) [41,43].

AD is an allergic disease with a chronic and recurrent course in which, as a result of mutations, e.g., impaired function and structure of the epidermal barrier are observed in the filaggrin gene [15,51]. In the course of AD, both quantitative and qualitative changes in the bacterial flora of the skin can occur. Numerous studies show that the microbiome of the skin of AD patients is significantly less diverse than that of healthy people [38,52]. A much smaller variety of bacteria is observed in the popliteal and elbow cavities. It is also important that the composition of the microbiome varies depending on the phase of the disease. Therefore, in periodic exacerbations of the disease, the dominance of *S. aureus* and *S. epidermidis* is observed, and in the period of remission—*Streptococcus, Propionibacterium* and *Corynebacterium* [53,54].

It should be emphasized that the presence of *S. aureus* within the skin is a very characteristic feature of AD, it has been observed that there are 10–100 times more of these bacteria compared to the healthy population [55]. The importance of this bacterium in the course of AD was described in the 1974 publication by Leyden et al. on the quantitative aspects of skin colonization of AD patients by *S. aureus*. According to the research of the above authors, the *S. aureus* colonization density within skin lesions exceeded 106 colony-forming units per cm^2^ [53,56]. Although the skin of patients with AD is almost always colonized by *S. aureus* with a tendency to develop overt infections, in patients with AD, an increase in the number of all staphylococcal strains is observed as well. Coagulase-negative strains show a protective effect through the ability to produce antibacterial proteins directed against *S. aureus*. They secrete phenol-soluble modulins, which damage the cell membrane of competing pathogens and stimulate keratinocytes to secrete natural proteins and lipoteichoic acid [57]. Whereas *S. aureus* produces exotoxins that have a negative effect on the skin [58].

On the surface of dendritic cells and keratinocytes, there are pattern recognition receptors that play a key role in recognizing pathogen-associated molecular patterns such as lipopolysaccharides, flagellin, Gram (-) bacterial nucleic acids; mannan and zymosan of fungi and peptidoglycans, lipoteichoic acid of Gram (+) bacteria. Activation of these receptors leads to the activation of the immune response as well as the accumulation of pro-inflammatory cytokines, chemokines and antibacterial proteins. In the acute phase of AD, it is observed that stimulated dendritic cells activate naive T cells in the regional lymph nodes, with the consequent proliferation of Th2 lymphocytes which return to the skin to produce pro-inflammatory cytokines; interleukins (IL4, IL5, IL13), this process causes the development of inflammation [57,59].

When it comes to fungal infections in AD, their role is not fully understood, it is known that *Malassezia* spp. can contribute to inflammation. According to studies, IgE antibodies directed against *Malassezia* spp. are observed in about 10–20% of AD patients [60]. It should be emphasized that the colonization by microorganisms consists in their presence on the skin surface without causing inflammatory changes; however, in certain situations, colonization may turn into infections with visible lesions, which also applies to AD patients [61]. 

Studies conducted in recent years indicate that the skin microbiota plays an extremely important role in protection against infections. Thus, the microbiota is crucial in maintaining healthy skin. However, it is known that there are numerous interactions between the skin and other organs. Changing the gut microbiome affects the immune system and thus the possibility of inflammation in other organs. There are studies confirming the relationship between intestinal dysbiosis and the development of inflammatory and immune diseases, including dermatological diseases [36,60,62].

## 4. The Role of Probiotics in Atopic Dermatitis

Probiotics are live microorganisms that, when consumed in proper amounts, are beneficial to the health of the host. Among the most well-known microorganisms that have probiotic activity bacteria from the genus, *Lactobacillus* and *Bifidobacterium* are distinguished. These are anaerobic, Gram (+) bacteria, belonging to the normal microbial flora. To be consumed by humans, probiotic microorganisms must be non-toxic and non-pathogenic [63,64,65]. Probiotics affect mainly the immune system and gastrointestinal tract by sealing the intestinal epithelium and inhibiting the penetration of allergens into the bloodstream [66,67]. The gut microbiome is a key regulator of postnatal immunity. Dysbiosis, meaning dysregulation of the microbiome that occurs particularly during the neonatal period, maybe a cofactor in the development of allergic disorders [68,69].

Over the past 20 years, there has been an increase in the number of studies that evaluate the use of probiotics for the treatment of children with AD and the prevention of AD in pregnant mothers [70]. Although the World Allergy Organization does not recommend the use of probiotics as a preventive measure against AD during pregnancy or while breastfeeding because there is no solid scientific evidence [71], a meta-analysis by Garcia-Larsen et al. which included 19 studies (over 4000 probands) showed that consumption of probiotics during the last period of pregnancy and breastfeeding reduced eczema (RR 0.78; 95% CI 0.68–0.90; I2 = 61%) or atopic eczema (RR 0.78; 95% CI 0.65–0.92; I2 = 0%)in children under 5 years of age allergic sensitization to cow’s milk between the ages of 1 and 2 years [72]. 

There are many studies on the effects of probiotics that have been conducted on a group of newborns. Zhao et al. conducted a meta-analysis of 8 clinical trials (741 infants), and the studies evaluated *Lactobacillus probiotics*. These probiotics have shown significant effects in reducing AD severity [73]. *Bifidobacterium probiotics* were used in a group of 73 infants. As the analysis shows, they did not show a positive effect. However, the study group was small, and in most studies, patients were observed for a short period of time, usually less than 8 weeks [74,75].

Research on the effects of probiotics is also thriving in children 1 to 18 years of age. A meta-analysis by Huang et al. considered 13 different studies. *L. fermentum*, *Lactobacillus* and a mixture of different strains (*B. bifidum, L. acidophilus, L. casei* and *L. salivarius*) significantly improved the severity scoring of atopic dermatitis (SCORAD) index values in children with AD. In contrast, *L. rhamnosus* and *L. plantarum* did not show any effectiveness on SCORAD values in children with AD [76]. *B. lactis* CECT 8145, *B. longum* CECT 7347, and *L. casei* CECT 9104 improved the skin appearance of AD patients aged 4 to 17 years (SCORAD index) in a study by Navarro-Lopez et al. [77].

Research studies have also been conducted in groups of adults with AD to test the effects of probiotics on alleviating skin lesions. Roessler et al. carried out a double-blind, placebo-controlled, randomized cross-over study. A group of 15 healthy subjects and 15 AD patients were included in the study. Probiotics (*L. paracasei* Lpc-37, *L. acidophilus* 74-2, and *B. animalis* ssp*. lactis* DGCC 420) or placebo were administered for 8 weeks. In patients with AD, SCORAD was used to determine the stage of the disease. After supplementation, increased faecal *L. paracasei* and *B. lactis* were detected in AD patients [78].

It is important to remember that commensal bacteria are one of the main factors that affect human health. Probiotics can manipulate the host microbiome and have a positive effect on reducing the effects of AD [79].

## 5. Conclusions

AD is a chronic skin disease with not fully elicitated aetiology and challenging treatment. Researchers are still searching for optimal management for patients with this skin condition. Studies on the microbiome, commensal microorganisms and the role of skin microorganisms in maintaining healthy skin bring additional insight into the treatment and prevention of AD. In the contemporary preventive and therapeutic approach to the management of the progression of atopic lesions, it is necessary to learn all the mechanisms determining pathogen-host dependence. An interesting perspective is given by the role of both innate and acquired resistance to microbes, which may translate into the degree of skin reaction to bacterial, fungal and yeast colonization of the skin. It seems that discovering all these relationships can contribute to the control of the course of the disease, prevent the aggravation of episodes of the disease and will positively affect the quality of life and activity of patients with atopic dermatitis in society. In the light of presented mechanisms, reduction in colonization may become both causative and symptomatic treatment in AD.

## Data Availability

Not applicable.

## References

[1] Rajka G. (1989). Essential Aspects of Atopic Dermatitis.

[2] Wallach D., Taïeb A. (2014). Atopic dermatitis/atopic eczema. Chem. Immunol. Allergy.

[3] Tilles G., Wallach D., Taïeb A. (2007). Topical therapy of atopic dermatitis: Controversies from Hippocrates to topical immunomodulators. J. Am. Acad. Dermatol..

[4] Dizon M.P., Yu A.M., Singh R.K., Wan J., Chren M.M., Flohr C., Silverberg J.I., Margolis D.J., Langan S.M., Abuabara K. (2018). Systematic review of atopic dermatitis disease definition in studies using routinely collected health data. Br. J. Dermatol..

[5] De Wit J., Totté J.E.E., Van Buchem F.J.M., Pasmans S. (2018). The prevalence of antibody responses against *Staphylococcus aureus* antigens in patients with atopic dermatitis: A systematic review and meta-analysis. Br. J. Dermatol..

[6] Totté J.E., van der Feltz W.T., Hennekam M., Van Belkum A., Van Zuuren E.J., Pasmans S.G. (2016). Prevalence and odds of *Staphylococcus aureus* carriage in atopic dermatitis: A systematic review and meta-analysis. Br. J. Dermatol..

[7] Benenson S., Zimhony O., Dahan D., Solomon M., Raveh D., Schlesinger Y., Yinnon A.M. (2005). Atopic dermatitis—A risk factor for invasive *Staphylococcus aureus* infections: Two cases and review. Am. J. Med..

[8] Baker B.S. (2006). The role of microorganisms in atopic dermatitis. Clin. Exp. Immunol..

[9] Lee K.S., Oh I.H., Choi S.H., Rha Y.H. (2017). Analysis of epidemiology and risk factors of atopic dermatitis in Korean children and adolescents from the 2010 Korean National Health and Nutrition Examination Survey. BioMed. Res. Int..

[10] Eichenfield L.F., Tom W.L., Chamlin S.L., Feldman S.R., Hanifin J.M., Simpson E.L., Berger T.G., Bergman J.N., Cohen D.E., Cooper K.D. (2014). Guidelines of care for the management of atopic dermatitis: Section 1. Diagnosis and assessment of atopic dermatitis. J. Am. Acad. Dermatol..

[11] Weidinger S., Novak N. (2016). Atopic dermatitis. Lancet.

[12] Czarnowicki T., Krueger J.G., Guttman-Yassky E. (2014). Skin barrier and immune dysregulation in atopic dermatitis: An evolving story with important clinical implications. J. Allergy Clin. Immunol. Pract..

[13] Agrawal R., Woodfolk J.A. (2014). Skin barrier defects in atopic dermatitis. Curr. Allergy Asthma Rep..

[14] Zeeuwen P.L.J.M., Boekhorst J., Van Den Bogaard E.H., De Koning H.D., Van De Kerkhof P.M.C., Saulnier D.M., Van Swam I.I., Van Hijum S.A.F.T., Kleerebezem M., Schalkwijk J. (2012). Microbiome dynamics of human epidermis following skin barrier disruption. Genome Biol..

[15] Palmer C.N., Irvine A.D., Terron-Kwiatkowski A., Zhao Y., Liao H., Lee S.P., Goudie D.R., Sandilands A., Campbell L.E., Smith F.J. (2006). Common loss-of-function variants of the epidermal barrier protein filaggrin are a major predisposing factor for atopic dermatitis. Nat. Genet..

[16] Le Lamer M., Pellerin L., Reynier M., Cau L., Pendaries V., Leprince C., Méchin M.C., Serre G., Paul C., Simon M. (2015). Defects of corneocyte structural proteins and epidermal barrier in atopic dermatitis. Biol. Chem..

[17] Norlén L., Al-Amoudi A. (2004). Stratum corneum keratin structure, function, and formation: The cubic rod-packing and membrane templating model. J. Investig. Dermatol..

[18] Jungersted J.M., Scheer H., Mempel M., Baurecht H., Cifuentes L., Høgh J.K., Hellgren L.I., Jemec G.B., Agner T., Weidinger S. (2010). Stratum corneum lipids, skin barrier function and filaggrin mutations in patients with atopic eczema. Allergy.

[19] Kezic S., Jakasa I. (2016). Filaggrin and Skin Barrier Function. Curr. Probl. Dermatol..

[20] Chan A., Terry W., Zhang H., Karmaus W., Ewart S., Holloway J.W., Roberts G., Kurukulaaratchy R., Arshad S.H. (2018). Filaggrin mutations increase allergic airway disease in childhood and adolescence through interactions with eczema and aeroallergen sensitization. Clin. Exp. Allergy.

[21] Ogrodowczyk A., Markiewicz L., Wróblewska B. (2014). Mutations in the filaggrin gene and food allergy. Przegladgastroenterologiczny.

[22] Candi E., Schmidt R., Melino G. (2005). The cornified envelope: A model of cell death in the skin. Nat. Rev. Mol. Cell Biol..

[23] Hon K.L., Leung A.K. (2013). Use of ceramides and related products for childhood-onset eczema. Recent Pat. Inflamm. Allergy Drug Discov..

[24] Elias P.K., Elias M.F., D’Agostino R.B., Sullivan L.M., Wolf P.A. (2005). Serum cholesterol and cognitive performance in the Framingham Heart Study. Psychosom. Med..

[25] Proksch E., Fölster-Holst R., Jensen J.M. (2006). Skin barrier function, epidermal proliferation and differentiation in eczema. J. Dermatol. Sci..

[26] Kenny J.G., Ward D., Josefsson E., Jonsson I.M., Hinds J., Rees H.H., Lindsay J.A., Tarkowski A., Horsburgh M.J. (2009). The *Staphylococcus aureus* response to unsaturated long chain free fatty acids: Survival mechanisms and virulence implications. PLoS ONE.

[27] Li S., Villarreal M., Stewart S., Choi J., Ganguli-Indra G., Babineau D.C., Philpot C., David G., Yoshida T., Boguniewicz M. (2017). Altered composition of epidermal lipids correlates with *Staphylococcus aureus* colonization status in atopic dermatitis. Br. J. Dermatol..

[28] Takigawa H., Nakagawa H., Kuzukawa M., Mori H., Imokawa G. (2005). Deficient production of hexadecenoic acid in the skin is associated in part with the vulnerability of atopic dermatitis patients to colonization by *Staphylococcus aureus*. Dermatology.

[29] Heczko P.B., Kasprowicz A., Kucharczyk J. (1971). Carrier state of *Staphylococcus aureus*. Susceptibility to free fatty acids of the representatives of the flora from the human nasal cavity. Med. Dosw. Mikrobiol..

[30] Magiatis P., Pappas P., Gaitanis G., Mexia N., Melliou E., Galanou M., Vlachos C., Stathopoulou K., Skaltsounis A.L., Marselos M. (2013). Malassezia yeasts produce a collection of exceptionally potent activators of the Ah (dioxin) receptor detected in diseased human skin. J. Investig. Dermatol..

[31] Naik S., Bouladoux N., Wilhelm C., Molloy M.J., Salcedo R., Kastenmuller W., Deming C., Quinones M., Koo L., Conlan S. (2012). Compartmentalized control of skin immunity by resident commensals. Science.

[32] Kubo A., Nagao K., Yokouchi M., Sasaki H., Amagai M. (2009). External antigen uptake by Langerhans cells with reorganization of epidermal tight junction barriers. J. Exp. Med..

[33] Blicharz L., Rudnicka L., Czuwara J., Waśkiel-Burnat A., Goldust M., Olszewska M., Samochocki Z. (2021). The Influence of Microbiome Dysbiosis and Bacterial Biofilms on Epidermal Barrier Function in Atopic Dermatitis-An Update. Int. J. Mol. Sci..

[34] Adamczyk K., Garncarczyk A.A., Antończak P.P. (2018). The microbiome of the skin. Dermatol. Rev..

[35] Grice E.A., Segre J.A. (2011). The skin microbiome. Nat. Rev. Microbiol..

[36] Belkaid Y., Naik S. (2013). Compartmentalized and systemic control of tissue immunity by commensals. Nat. Immunol..

[37] Findley K., Oh J., Yang J., Conlan S., Deming C., Meyer J.A., Schoenfeld D., Nomicos E., Park M., Becker J. (2013). Topographic diversity of fungal and bacterial communities in human skin. Nature.

[38] Kong H.H., Oh J., Deming C., Conlan S., Grice E.A., Beatson M.A., Nomicos E., Polley E.C., Komarow H.D., Program N.C.S. (2012). Temporal shifts in the skin microbiome associated with disease flares and treatment in children with atopic dermatitis. Genome Res..

[39] Percival S.L., Emanuel C., Cutting K.F., Williams D.W. (2012). Microbiology of the skin and the role of biofilms in infection. Int. Wound J..

[40] Kong H.H. (2011). Skin microbiome: Genomics-based insights into the diversity and role of skin microbes. Trends Mol. Med..

[41] Kong H.H., Andersson B., Clavel T., Common J.E., Jackson S.A., Olson N.D., Segre J.A., Traidl-Hoffmann C. (2017). Performing skin microbiome research: A method to the madness. J. Investig. Dermatol..

[42] Rieg S., Garbe C., Sauer B., Kalbacher H., Schittek B. (2004). Dermcidin is constitutively produced by eccrine sweat glands and is not induced in epidermal cells under inflammatory skin conditions. Br. J. Dermatol..

[43] Oh J., Byrd A.L., Park M., Kong H.H., Segre J.A. (2016). Temporal stability of the human skin microbiome. Cell.

[44] Rahim K., Saleha S., Zhu X., Huo L., Basit A., Franco O.L. (2017). Bacterial contribution in chronicity of wounds. Microb. Ecol..

[45] Grice E.A., Kong H.H., Conlan S., Deming C.B., Davis J., Young A.C., Program N.C.S., Bouffard G.G., Blakesley R.W., Murray P.R. (2009). Topographical and temporal diversity of the human skin microbiome. Science.

[46] Schommer N.N., Gallo R.L. (2013). Structure and function of the human skin microbiome. Trends Microbiol..

[47] Gaitanis G., Magiatis P., Hantschke M., Bassukas I.D., Velegraki A. (2012). The Malassezia genus in skin and systemic diseases. Clin. Microbiol. Rev..

[48] Glatz M., Bosshard P.P., Hoetzenecker W., Schmid-Grendelmeier P. (2015). The Role of *Malassezia* spp. in Atopic Dermatitis. J. Clin. Med..

[49] Jagielski T., Rup E., Macura A.B., Bielecki J. (2013). Characterization of fungi of the Malassezia genus. I. Microbiological and immunological aspect. Postepy Mikrobiol..

[50] Bikowski J.B., Del Rosso J.Q. (2009). Demodex dermatitis: A retrospective analysis of clinical diagnosis and successful treatment with topical crotamiton. J. Clin. Aesthet. Dermatol..

[51] Kasznia-Kocot J., Reichmann K., Wypych-Ślusarska A. (2014). Selected aspects of quality of life in atopic dermatitis. Medycyna Środowiskowa.

[52] Kennedy E.A., Connolly J., Hourihane J.O.B., Fallon P.G., McLean W.H.I., Murray D., Jo J.-H., Segre J.A., Kong H.H., Irvine A.D. (2017). Skin microbiome before development of atopic dermatitis: Early colonization with commensal staphylococci at 2months is associated with a lower risk of atopic dermatitis at 1year. J. Allergy Clin. Immunol..

[53] Hasse-Cieślińska M. (2007). *Staphylococcus aureus* skin colonization in atopic dermatitis patients. Post Dermatol. Alergol..

[54] Kaczmarek J., Kuna P. (2015). The role of the microbiome in the development of allergies—Can we modify it?. Terapia.

[55] Gloor M., Peters G., Stoika D. (1982). On the resident aerobic bacterial skin flora in unaffected skin of patients with atopic dermatitis and in healthy controls. Dermatologica.

[56] Leyden J.J., Marples R.R., Kligman A.M. (1974). *Staphylococcus aureus* in the lesions of atopic dermatitis. Br. J. Dermatol..

[57] Narbutt J. (2019). The role of the microbiome in atopic dermatitis. Pediatria Dyplomie..

[58] Cogen A.L., Yamasaki K., Sanchez K.M., Dorschner R.A., Lai Y., MacLeod D.T., Torpey J.W., Otto M., Nizet V., Kim J.E. (2010). Selective antimicrobial action is provided by phenol-soluble modulins derived from *Staphylococcus epidermidis*, a normal resident of the skin. J. Investig. Dermatol..

[59] Meng J., Li Y., Fischer M.J.M., Steinhoff M., Chen W., Wang J. (2021). Th2 Modulation of Transient Receptor Potential Channels: An Unmet Therapeutic Intervention for Atopic Dermatitis. Front. Immunol..

[60] Darabi K., Hostetler S.G., Bechtel M.A., Zirwas M. (2009). The role of Malassezia in atopic dermatitis affecting the head and neck of adults. J. Am. Acad. Dermatol..

[61] Nowicka D., Nawrot U. (2019). Contribution of *Malassezia* spp. to the development of atopic dermatitis. Mycoses.

[62] Wakkee M., Nijsten T. (2009). Comorbidities in dermatology. Dermatol. Clin..

[63] Hill C., Guarner F., Reid G., Gibson G.R., Merenstein D.J., Pot B., Morelli L., Canani R.B., Flint H.J., Salminen S. (2014). Expert consensus document. The International Scientific Association for Probiotics and Prebiotics consensus statement on the scope and appropriate use of the term probiotic. Nat. Rev. Gastroenterol. Hepatol..

[64] Rusu E., Enache G., Cursaru R., Alexescu A., Radu R., Onila O., Cavallioti T., Rusu F., Posea M., Jinga M. (2019). Prebiotics and probiotics in atopic dermatitis. Exp.Ther. Med..

[65] Fanfaret I.S., Boda D., Ion L.M., Hosseyni D., Leru P., Ali S., Corcea S., Bumbacea R. (2021). Probiotics and prebiotics in atopic dermatitis: Pros and cons (Review). Exp.Ther. Med..

[66] Martins A.K., Martins F.S., Gomes D.A., Elian S.D., Vieira A.T., Teixeira M.M., Cara D.C., Nardi R.M., Nicoli J.R. (2010). Evaluation of in vitro antagonism and of in vivo immune modulation and protection against pathogenic experimental challenge of two probiotic strains of Bifidobacterium animalis var. lactis. Arch. Microbiol..

[67] Ohland C.L., Macnaughton W.K. (2010). Probiotic bacteria and intestinal epithelial barrier function. Am. J. Physiol.Gastrointest. Liver Physiol..

[68] Dominguez-Bello M.G., Godoy-Vitorino F., Knight R., Blaser M.J. (2019). Role of the microbiome in human development. Gut.

[69] Gerlich J., Benecke N., Peters-Weist A.S., Heinrich S., Roller D., Genuneit J., Weinmayr G., Windstetter D., Dressel H., Range U. (2018). Pregnancy and perinatal conditions and atopic disease prevalence in childhood and adulthood. Allergy.

[70] Kramer M.S., Kakuma R. (2012). Maternal dietary antigen avoidance during pregnancy or lactation, or both, for preventing or treating atopic disease in the child. Cochrane Database Syst. Rev..

[71] Fiocchi A., Pawankar R., Cuello-Garcia C., Ahn K., Al-Hammadi S., Agarwal A., Beyer K., Burks W., Canonica G.W., Ebisawa M. (2015). World Allergy Organization-McMaster University Guidelines for Allergic Disease Prevention (GLAD-P): Probiotics. World Allergy Organ J..

[72] Garcia-Larsen V., Ierodiakonou D., Jarrold K., Cunha S., Chivinge J., Robinson Z., Geoghegan N., Ruparelia A., Devani P., Trivella M. (2018). Diet during pregnancy and infancy and risk of allergic or autoimmune disease: A systematic review and meta-analysis. PLoS Med..

[73] Zhao M., Shen C., Ma L. (2018). Treatment efficacy of probiotics on atopic dermatitis, zooming in on infants: A systematic review and meta-analysis. Int. J. Dermatol..

[74] Björkstén B. (2004). Effects of intestinal microflora and the environment on the development of asthma and allergy. Springer Semin. Immunopathol..

[75] Murray C.S., Tannock G.W., Simon M.A., Harmsen H.J., Welling G.W., Custovic A., Woodcock A. (2005). Fecal microbiota in sensitized wheezy and non-sensitized non-wheezy children: A nested case-control study. Clin. Exp. Allergy.

[76] Huang R., Ning H., Shen M., Li J., Zhang J., Chen X. (2017). Probiotics for the treatment of atopic dermatitis in children: A systematic review and meta-analysis of randomized controlled trials. Front. Cell Infect. Microbiol..

[77] Navarro-López V., Ramírez-Boscá A., Ramón-Vidal D., Ruzafa-Costas B., Genovés-Martínez S., Chenoll-Cuadros E., Carrión-Gutiérrez M., Horga de la Parte J., Prieto-Merino D., Codoñer-Cortés F.M. (2018). Effect of oral administration of a mixture of probiotic strains on SCORAD Index and use of topical steroids in young patients with moderate atopic dermatitis: A randomized clinical trial. JAMA Dermatol..

[78] Roessler A., Friedrich U., Vogelsang H., Bauer A., Kaatz M., Hipler U.C., Schmidt I., Jahreis G. (2008). The immune system in healthy adults and patients with atopic dermatitis seems to be affected differently by a probiotic intervention. Clin. Exp. Allergy.

[79] Yu Y., Dunaway S., Champer J., Kim J., Alikhan A. (2020). Changing our microbiome: Probiotics in dermatology. Br. J. Dermatol..

